# Development of the nonreceptor tyrosine kinase FER-targeting PROTACs as a potential strategy for antagonizing ovarian cancer cell motility and invasiveness

**DOI:** 10.1016/j.jbc.2023.104825

**Published:** 2023-05-16

**Authors:** Yanchun Zhang, Xuexue Xiong, Renhong Sun, Xiaotong Zhu, Chen Wang, Biao Jiang, Xiaobao Yang, Dake Li, Gaofeng Fan

**Affiliations:** 1 School of Life Science and Technology, ShanghaiTech University, Shanghai, China; 2 Gluetacs Therapeutics (Shanghai) Co, Ltd, Shanghai, China; 3 Shanghai Institute for Advanced Immunochemical Studies, ShanghaiTech University, Shanghai, China; 4 Department of Gynecology, Women’s Hospital of Nanjing Medical University, Nanjing Maternity and Child Health Care Hospital, Nanjing, China

**Keywords:** protein degradation, PROTAC, tyrosine kinase, FER, ovarian cancer

## Abstract

Aberrant overexpression of nonreceptor tyrosine kinase FER (Fps/Fes Related) has been reported in various ovarian carcinoma–derived tumor cells and is a poor prognosis factor for patient survival. It plays an essential role in tumor cell migration and invasion, acting concurrently in both kinase-dependent and -independent manners, which is not easily suppressed by conventional enzymatic inhibitors. Nevertheless, the PROteolysis-TArgeting Chimera (PROTAC) technology offers superior efficacy over traditional activity–based inhibitors by simultaneously targeting enzymatic and scaffold functions. Hence in this study, we report the development of two PROTAC compounds that promote robust FER degradation in a cereblon-dependent manner. Both PROTAC degraders outperform a Food and Drug Administration–approved drug, brigatinib, in ovarian cancer cell motility suppression. Importantly, these PROTAC compounds also degrade multiple oncogenic FER fusion proteins identified in human tumor samples. These results lay an experimental foundation to apply the PROTAC strategy to antagonize cell motility and invasiveness in ovarian and other types of cancers with aberrant expression of FER kinase and highlight PROTACs as a superior strategy for targeting proteins with multiple tumor-promoting functions.

Ovarian cancer is one of the most malignant gynecological cancers, with high morbidity and mortality among women (1). Despite substantial advances in surgery and chemical- and radiation therapy for ovarian cancer, medical challenges such as metastasis and resistance remain unresolved. An in-depth understanding of ovarian tumor progression and metastasis will be critical in identifying new therapeutic targets to intervene in this heterogeneous and lethal disease (2).

Tyrosine phosphorylation, precisely coordinated by tyrosine kinases and phosphatases, regulates multilayer signaling networks spatiotemporal dependently. Feline sarcoma (FES)–related kinase FER (Fps/Fes Related), along with FES, represents a distinct nonreceptor tyrosine kinase subfamily characterized by a functional N-terminal membrane-targeting F-BAR domain, a central SH2 domain, and a C-terminal kinase domain (3) with essential roles in cell proliferation, motility, intercellular adhesion as well as the mediation of signal transmission from the cell surface to the cytoskeleton (3, 4, 5). Previous studies have strongly suggested that aberrantly high expression of FER as an independent prognostic indicator (6, 7) is associated with tumor progression (8, 9, 10, 11, 12, 13, 14, 15) and metastasis (6, 16, 17, 18) in several cancer types. In particular, FER is significantly upregulated in ovarian carcinoma samples and carcinoma-derived cell lines. Downregulation of FER substantially inhibits tumor cell migration, invasion, and metastasis (19, 20), indicating the urgent need and market potential for developing antagonists against FER kinase to benefit ovarian cancer patients. So far, only one small-molecule inhibitor targeting the kinase domain of FER has been reported (21). Of note, several studies also revealed the kinase-independent function of FER in regulating cell motility (20, 21, 22, 23). Therefore, targeting kinase activity alone with the conventional inhibitor seems to need improvement to block the whole spectrum of the enzyme’s function.

Recently, we have witnessed revolutionary paradigm shifts in the landscape of drug design from traditional enzymatic inhibition strategy to more challenging small-molecule–induced protein degradation technology. PROTAC (PROteolysis-TArgeting Chimera), as one of the cores of this technology, is a bifunctional small-molecule compound with one ligand that binds to the target protein and another ligand that binds to E3 ubiquitin ligase, with a linker in between, which allows target protein to irreversibly enter the ubiquitin–proteasome pathway for degradation to affect all functions of the protein (24, 25, 26, 27, 28). Compared with small-molecule inhibitors and macromolecular antibodies, PROTACs have many distinct advantages, including low dosage, low toxicity, and high selectivity (26). Of most importance, PROTAC technology can expand its client reservoir to traditionally undruggable targets and overcome drug resistance caused by mutation or overexpression (20, 27). Furthermore, this protein degradation–oriented strategy could simultaneously eliminate the target's enzymatic-dependent and -independent functions (29), resulting in complete inhibition and a lower chance for acquired resistance.

Given the critical role of FER in ovarian cancer and both kinase-dependent and -independent functions (20), we aimed to employ protein degradation technology to design a PROTAC degrader of FER and conduct an in-depth activity evaluation and mechanism study of this compound. This will lay a solid experimental foundation for the ultimate development of the FER-targeting PROTAC drug. Meanwhile, it will also provide substantial evidence for supporting FER as an essential target for ovarian cancer and the scientific significance of degrading FER protein for treating ovarian cancer patients.

## Results

### Design and synthesis of FER-targeting PROTAC compounds

**Figure 1:**
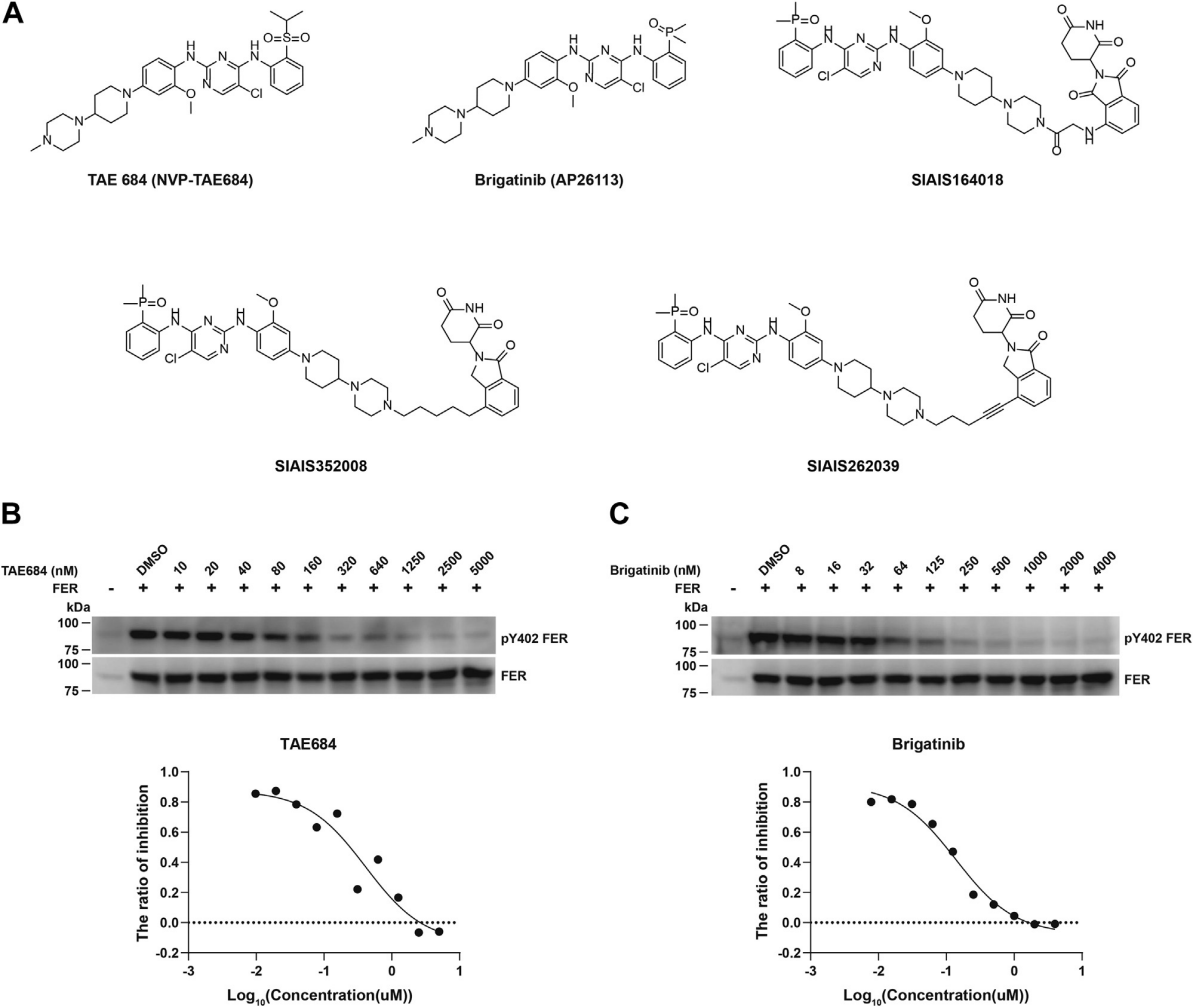
**Design and synthesis of FER-targeting PROTAC compounds.***A*, structure of TAE684, brigatinib, SIAIS164018, SIAIS352008, and SIAIS262039. *B* and *C*, HEK293FT cells were transiently transfected with pLV-FER, followed by TAE684 (*B*) or brigatinib (*C*) treatment for 16 h, as indicated. The phosphorylation level of tyrosine 402 of FER was detected by immunoblotting analysis with an anti-pY402-FER antibody. Total FER in whole-cell lysate samples was also probed. IC_50_ was calculated with dose–response nonlinear regression drawn by GraphPad Prism. FER, Fps/Fes Related; HEK293FT, human embryonic kidney 293FT cell line; PROTAC, PROteolysis TArgeting Chimera.

TAE684 is a small-molecule compound screened as the first-generation inhibitor of anaplastic lymphoma kinase (ALK) (Fig. 1*A*) (30). Interestingly, it also exhibits inhibitory activity against FES kinase, another member of the FER kinase family (31). However, TAE684 fails to enter clinical research because of its potential oxidative and metabolic toxicity (30, 32). The emergence of drug resistance and increased demand for better medicines have led to the development of second- and third-generation ALK inhibitors (33). Brigatinib (Fig. 1*A*) has been approved for treating ALK-positive metastatic non–small cell lung cancer that has deteriorated after crizotinib treatment or is intolerant to crizotinib in 2017 as for the first-line treatment of ALK-positive metastatic non–small cell lung cancer in 2020. Meanwhile, the KINOSMEScan profiling showed that brigatinib has a strong binding affinity to FER, second only to ALK (34). As a starting point of the project, we assessed the inhibitory effect of TAE684 and brigatinib on FER kinase activity by monitoring the phosphorylation of Tyr402, one of its autophosphorylation sites, upon activation. Indeed, both TAE684 and brigatinib effectively inhibited the phosphorylation of FER at Tyr402, with IC_50_ reaching 0.4106 and 0.1375 μM, respectively (Fig. 1, *B* and *C*). According to the value of IC_50_, both brigatinib and TAE684 exhibited a superior inhibitory effect on the kinase activity of FER than E260 (21), the only reported FER kinase inhibitor with IC_50_ around 2 μM.

In pursuing PROTAC compounds that degrade ALK, with brigatinib as the warhead, we consistently noticed that one of the lead compounds, SIAIS164018 (Fig. 1*A*), could degrade ALK and FER simultaneously (35). SIAIS164018 consisted of demethylated brigatinib as ALK or FER binder, pomalidomide as cereblon (CRBN) E3 ligase ligand, and a short acetyl linker. Interestingly, the KINOMEScan profiling revealed that SIAIS164018 inhibited FER kinase more preferentially than ALK (35). Nevertheless, the degradation capability of SIAIS164018 on FER still needs to be improved (35). After multiple rounds of a structure optimization, including using lenalidomide to replace pomalidomide and screening the length and type of linkers, we finally obtained SIAIS352008 and SIAIS262039 (hereinafter referred to as 008 and 039, Fig. 1*A*) with better degradation efficacy. We evaluated their biochemical and biological properties in the context of ovarian cancer.

### PROTAC compounds effectively degrade FER kinase

**Figure 2:**
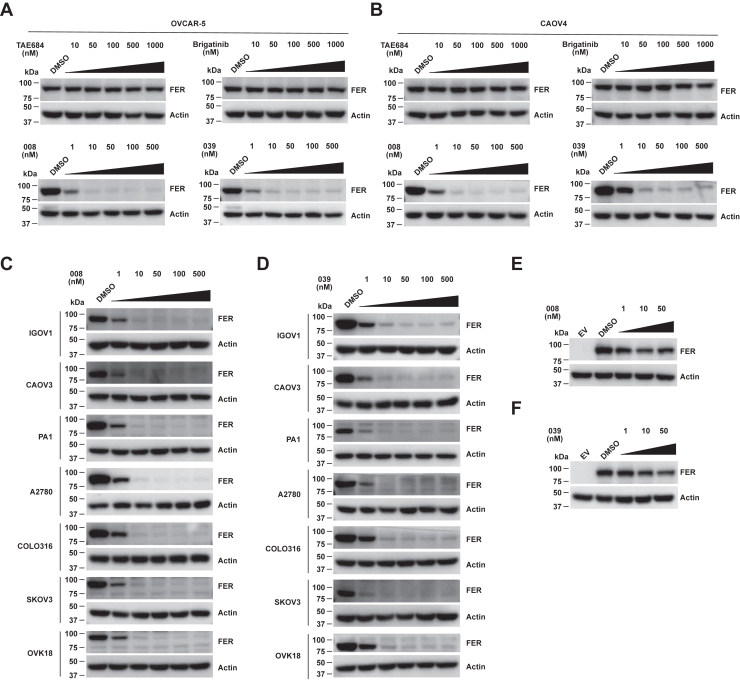
**PROTAC compounds effectively degrade FER kinase in multiple ovarian cancer cell lines.***A* and *B*, OVCAR-5 (*A*) and CAOV4 (*B*) cells were treated with TAE684, brigatinib, 008, or 039, respectively, for 16 h. Lysates were harvested, and the expressions of FER were detected by immunoblotting analysis, with actin as a loading control. *C* and *D*, seven ovarian cancer cells with FER expression were treated with compounds 008 (*C*) or 039 (*D*) for 16 h, respectively. Lysates were harvested, and the expressions of FER were detected by immunoblotting analysis, with actin as a loading control. *E* and *F*, after transient transfection with pLV-FER, HEK293FT cells were treated with compounds 008 or 039 for 16 h, followed by immunoblotting analysis with FER and actin. FER, Fps/Fes Related; HEK293FT, human embryonic kidney 293FT cell line; PROTAC, PROteolysis TArgeting Chimera.

Previous studies have demonstrated the upregulation of FER proteins in multiple ovarian carcinoma–derived cell lines (20). We evaluated the protein degradation efficacy of PROTAC compounds 008 and 039 first on OVCAR-5 and CAOV4 ovarian cancer cell lines, which have the highest FER protein expression (20). Compared with the dimethyl sulfoxide (DMSO) control, 008 or 039 treatment robustly degraded the kinase, and the effective concentration was as low as 1 nM (Fig. 2, *A* and *B*). In addition, TAE684 and brigatinib demonstrated no degradation capability of FER kinase even at a high concentration of 1000 nM (Fig. 2, *A* and *B*). Next, we assessed whether the 008- and 039-mediated dynamic FER protein turnover could be recapitulated in the other seven ovarian cancer cell lines with high FER expression. Indeed, both PROTAC compounds universally disrupt the endogenous FER proteins in these cell lines (Fig. 2, *C* and *D*). Third, we evaluated the capability of these two compounds to degrade the ectopically overexpressed FER proteins in human embryonic kidney 293FT (HEK293FT) cells. As shown in Figure 2, *E* and *F*, both 008 and 039 could also degrade the exogenous FER protein in a dose-dependent manner. In summary, these two PROTAC compounds effectively disrupted the expression of FER proteins in cells.

**Figure 3:**
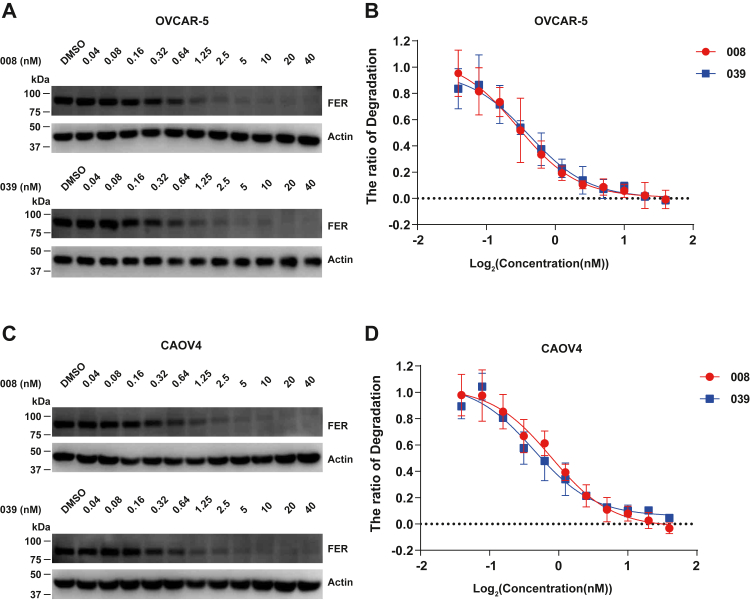
**DC**_**50**_**measurement of the PROTAC compounds in OVCAR-5 and CAOV4 ovarian cancer cell lines.** OVCAR-5 (*A* and *B*) and CAOV4 (*C* and *D*) cells were treated with compounds 008 or 039 for 16 h, as indicated. *A* and *C*, the protein level of FER was detected by immunoblotting analysis with an FER antibody. Actin was also probed as a loading control. *B* and *D*, results were represented as means ± SD from three independent replicates. DC_50_ was calculated with dose–response nonlinear regression drawn by GraphPad Prism. FER, Fps/Fes Related; PROTAC, PROteolysis TArgeting Chimera.

To accurately quantify the efficacy of both PROTAC compounds on FER protein degradation, we measured their DC_50_ values in OVCAR-5 and CAOV4 cell lines. The results indicated that 008 and 039 degraded endogenous FER protein in a concentration-dependent manner without any sign of hook effect (Fig. 3, *A* and *C*). The DC_50_ values of 008 and 039 in the OVCAR-5 cell line were 0.2883 nM and 0.4113 nM, respectively (Fig. 3*B*), and in the CAOV4 cell line, were 0.7839 nM and 0.4356 nM, respectively (Fig. 3*D*).

### PROTAC compounds degrade FER in a cereblon- and proteasome-dependent manner

**Figure 4:**
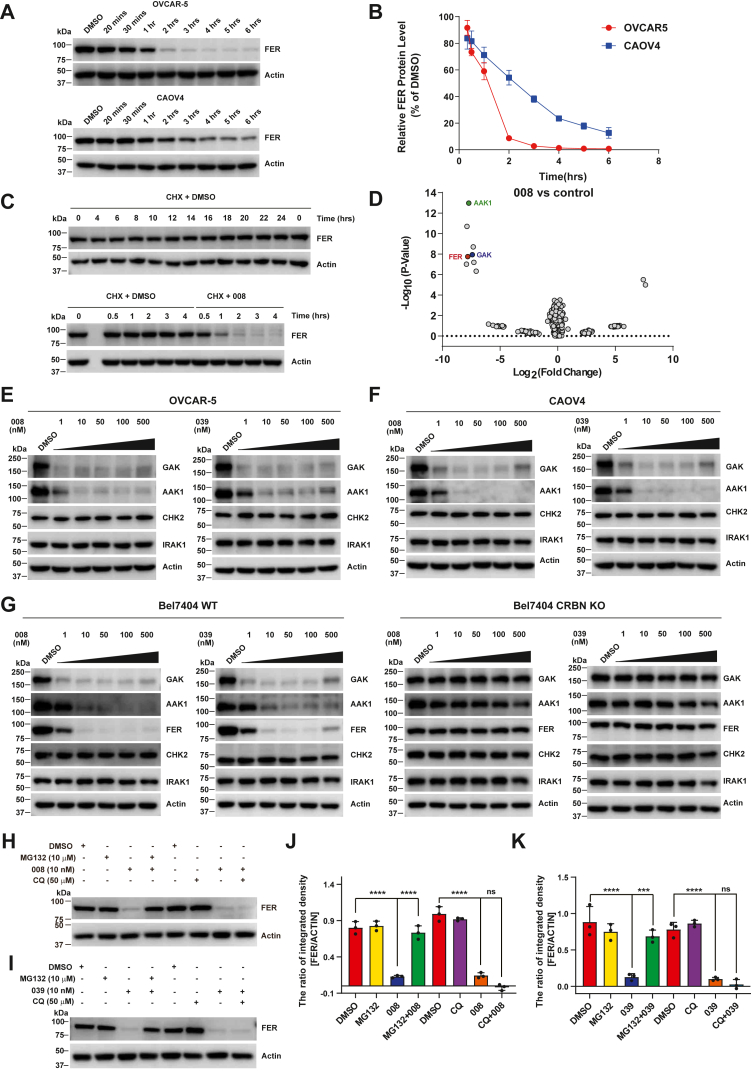
**PROTAC compounds degrade FER in a cereblon- and proteasome-dependent manner.***A*, OVCAR-5 cells or CAOV4 cells were treated with 10 nM 008, respectively, and harvested at the indicated time points for immunoblotting analysis. Total FER and actin were probed. *B*, results from (*A*) were represented as means ± SD from three independent replicates. The time–response nonlinear regression was drawn by GraphPad Prism. *C*, OVCAR-5 cells, in the presence of 10 μg/ml CHX, were treated with DMSO or 100 nM 008, followed by immunoblotting analysis. Total FER and actin were probed. *D*, OVCAR-5 cells were treated with DMSO or compound 008 for 16 h and then subjected to mass spectrometry analysis. Each experiment was repeated three times. *E* and *F*, OVCAR-5 and CAOV4 cells were treated with 008 or 039, respectively, for 16 h. The protein levels of GAK and AAK1 were detected by the corresponding antibodies, as indicated. Expressions of negative control CHK2 and IRAK1 were also probed, with actin as a loading control. *G*, Bel7404^WT^ and Bel7404^CRBN−/−^ knockout cell lines were treated with 008 and 039 for 16 h. The protein levels of FER, GAK, AAK1, CHK2, IRAK1, and loading control actin were checked with immunoblotting analysis. *H*–*K*, OVCAR-5 cells were treated with the proteasome inhibitor MG132 or the lysosome inhibitor CQ in combination with compounds 008 (*H*) or 039 (*I*) for 4 h, as indicated. Cell lysates were harvested and immunoblotted with an FER antibody, using actin as a loading control. Results represented means ± SD from three replicates (*J* and *K*). ∗∗∗*p* < 0.001 and ∗∗∗∗*p* < 0.0001. AAK1, AP2-associated kinase 1; CHX, cycloheximide; DMSO, dimethyl sulfoxide; FER, Fps/Fes Related; GAK, cyclin G-associated kinase; PROTAC, PROteolysis TArgeting Chimera.

To explore the time trajectory of endogenous FER degradation by PROTAC compounds, we treated OVCAR-5 or CAOV4 cells with 10 nM 008, respectively, and collected samples at indicated time points for immunoblotting analysis. The PROTAC 008 started to degrade FER proteins in OVCAR-5 cells 20 min after treatment, and the degradation reached the maximum level in 3 h. The FER protein in CAOV4 cells began to be degraded 30 min after the treatment and reached the maximum degradation in 6 h, respectively (Fig. 4, *A* and *B*).

Meanwhile, we applied the cycloheximide (CHX) treatment to estimate the half-life of the FER proteins in OVCAR-5 cells. The kinase was relatively stable in the DMSO group, with no significant degradation occurring in 24 h (Fig. 4*C*). However, the expression level of FER proteins was significantly reduced upon 100 nM 008 treatment, with a half-life of less than 1 h (Fig. 4*C*). These results indicated that the PROTAC compounds' addition largely destroyed the FER protein's stability.

To evaluate the degradation selectivity of our PROTAC compound, we performed a mass spectrometry–based quantitative proteomics analysis in the OVCAR-5 cell line in the absence and presence of the 008 compounds. A total of 4458 proteins were identified by mass spectrometry analysis, among which eight proteins could be degraded by 008 in three independent repeated experiments (AAK1 [AP2-associated kinase 1], BTF3 [basic transcription factor 3], PARS2 (prolyl-TRNA synthetase 2), GAK (cyclin G-associated kinase), FER, YEATS2 (YEATS domain containing 2), DRAM2 (DNA damage–regulated autophagy modulator 2), and PPIL2 [peptidylprolyl isomerase like 2]), as illustrated by the volcano plot (Fig. 4*D*). It is worth noting that AAK1, GAK, and FER are kinases. Compared with DMSO-treated control samples, 008-treated cells showed a 99.56% (*p* = 1.776 × 10^−8^) reduction in FER protein level (Fig. 4*D*).

Subsequently, we verified the top candidate kinases shown in the high-confidence intervals of the volcano plot. The immunoblotting analysis confirmed that AAK1, GAK, and FER protein levels in OVCAR-5 and CAOV4 cells were significantly downregulated upon 008 or 039 treatment for 16 h (Fig. 4, *E* and *F*). IRAK1 and CHK2, two negative control kinases we included, showed no protein level change in the same treatment condition (Fig. 4, *E* and *F*). These data indicated that the downregulation of AAK1, GAK, and FER induced by 008 and 039 is selective.

Two major systems within cells are responsible for protein quality control: the ubiquitin–proteasome system (UPS) and the lysosome-mediated protein degradation system (36). Usually, the PROTAC technology harnesses the UPS system for target protein clearance (25). Hence, we attempted to determine whether 008- or 039-induced FER degradation depends on the UPS route. As shown in Figure 4*G*, 008 triggered robust degradation of FER as well as AAK1 and GAK1 in the liver carcinoma cell line Bel7404; however, CRISPR–Cas9-meditated E3 ligase CRBN knockout significantly blocked turnover of these kinase proteins, indicating that the E3 ligase CRBN is required for PROTAC compound–mediated kinase degradation. Adding the proteasome inhibitor MG132, but not lysosome inhibitor chloroquine, effectively prevented the degradation of FER protein initiated by these two compounds (Fig. 4, *H*–*K*), confirming the necessity of proteasome machinery in FER clearance.

### PROTAC compounds suppress ovarian cancer cell motility and invasiveness

Given the superior degradation effect on FER kinase, we conducted a series of cell-based assays to assess their anticancer properties. CTG-based cell proliferation assay showed no significant growth delay upon treating PROTACs in four ovarian cancer cell lines, including CAOV4, OVK18, PA-1, and COLO316 (Fig. S1, *A*–*D*). Consistently, no significant difference was observed neither in cell cycle distribution (Fig. S1, *E*–*F*) nor in cell apoptosis (Fig. S1, *G*–*H*) between DMSO or PROTAC-treated CAOV4 cells, indicating these compounds have minimal capability in inhibiting ovarian cancer cell proliferation and survival.

**Figure 5:**
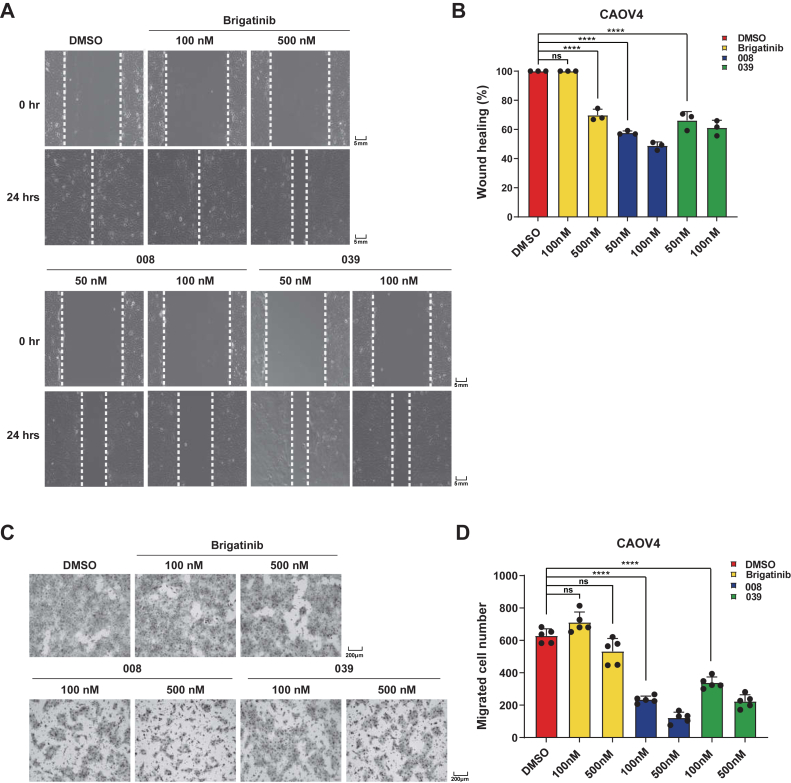
**PROTAC compounds suppress ovarian cancer cell motility and invasiveness.***A* and *B*, CAOV4 cells were treated with DMSO, brigatinib, or PROTACs, respectively, followed by the wound healing experiment. *A*, wound widths were measured, and wound closure rates were calculated. *B*, results were represented as means ± SD from three independent replicates. ∗∗∗∗*p* < 0.0001. *C* and *D*, CAOV4 cells were treated with DMSO, brigatinib, or PROTACs, respectively, followed by the Boyden chamber migration assay (*C*). Results were represented as means ± SD from five independent replicates (*D*). ∗∗∗∗*p* < 0.0001. DMSO, dimethyl sulfoxide; PROTAC, PROteolysis TArgeting Chimera.

Cancer metastasis often leads to treatment failure. The FER kinase has been reported to regulate cell migration and invasion in many types of cancers (6, 16, 17, 18). To evaluate the efficacy of the FER-targeting PROTAC compounds on ovarian cancer cell motility suppression, we performed the wound healing assay in CAOV4 cells, using brigatinib as a control. As shown in Figure 5, *A* and *B*, whereas brigatinib exhibited mild suppression at a high concentration (500 nM), both 008 and 039 inhibited cell wound healing to a greater extent at a relatively low concentration (50 and 100 nM). Indeed, this inhibitory effect on cell motility was ready to be observed at 10 nM (Fig. S2, *A* and *B*), the concentration of which caused effective FER protein degradation. Consistently, we observed similar results in the transwell assay. Compared with brigatinib, which exhibited slight inhibition on cell migration, 008 and 039 significantly inhibited cell migration through the Boyden chamber cassette at both low (100 nM) and high (500 nM) concentrations (Fig. 5, *C* and *D*). Actually, we started to observe a significant delay in cell migration from 10 nM compound concentration (Fig. S2, *C* and *D*). Therefore, both PROTAC compounds demonstrated superior function than brigatinib on ovarian cancer cell motility suppression.

**Figure 6:**
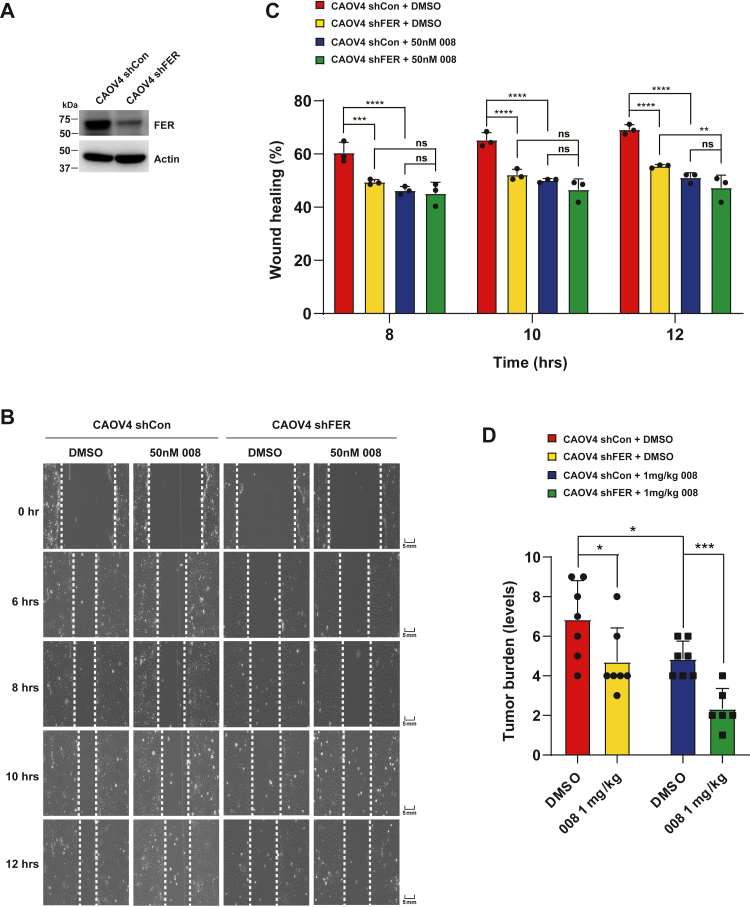
**PROTAC-mediated FER degradation majorly accounts for decreased ovarian cancer cell motility and invasiveness.***A*, cell lysates were harvested from the CAOV4^shCon^ and CAOV4^shFER^ cells and immunoblotted with an FER antibody, using actin as a loading control. *B* and *C*, CAOV4^shCon^ and CAOV4^shFER^ cells were treated with DMSO or 50 nM compound 008, followed by the wound healing experiment at indicated time points (*B*). Results were represented as means ± SD from three independent replicates (*C*). ∗∗*p* < 0.01; ∗∗∗*p* < 0.001; ∗∗∗∗*p* < 0.0001. *D*, the effects of PROTAC compound 008 on xenograft NSG models by intraperitoneal administration. Three million CAOV4^shCon^ or CAOV4^shFER^ cells were injected into each mouse, followed by an intraperitoneal injection of 008 at a 1 mg/kg dose every 3 days. Mice were sacrificed 5 weeks after injection. Cells only colonized on the peritoneum. Tumor burden was graded from 0 to 10. Bars show the mean ± SD of tumor burden from seven donors of the shCon + DMSO group, seven donors of the shCon + 008 group, seven donors of the shFER + DMSO group, and six donors of the shFER + 008 group. ∗*p* < 0.05; ∗∗∗*p* < 0.001. DMSO, dimethyl sulfoxide; FER, Fps/Fes Related; PROTAC, PROteolysis TArgeting Chimera.

To verify that the PROTAC-induced cell motility decrease is due to the degradation of FER protein, we established a FER-knockdown CAOV4 cell line using shRNA technology (Fig. 6*A*), followed by the wound healing assay. Indeed, the wound-healing ability of CAOV4^shFER^ cells was lower than that of CAOV4^shCon^ cells (Fig. 6, *B* and *C*). We also observed that 008 significantly dampened the wound-healing capacity of CAOV4 cells (Fig. 6, *B* and *C*). Interestingly, there was little difference in the migration ability of 008-treated CAOV4^shCon^ and CAOV4^shFER^ cells up to 12 h of treatment, indicating the suppressive effect of PROTAC compounds was due to the on-target degradation of FER protein, at least under the current experimental conditions (Fig. 6, *B* and *C*).

To further evaluate the efficacy of these two PROTAC compounds *in vivo*, we intraperitoneally injected CAOV4^shCon^ or CAOV4^shFER^ cells in NSG mice, followed by either vehicle or 008 treatment and counted the number of tumor nodules of metastasis on the peritoneal wall. Compared with the mice injected with CAOV4^shCon^ cells, the pharmacological degradation of FER kinase or the genetical knockdown of FER expression showed an equivalent decrease in tumor nodules on the peritoneal wall (Fig. 6*D*). Interestingly, FER knockdown synergized with the administration of the PROTAC degrader 008 to show a more reduced number of tumor nodules on the peritoneal wall, implying either incomplete suppression of FER expression by shRNA or the potential degradation of other targets may also contribute to the reduced metastasis phenotype (Fig. 6*D*). Nevertheless, the PROTAC compound 008 significantly suppressed ovarian cancer cell motility and invasiveness *in vivo*.

### PROTAC compounds degrade oncogenic FER fusion proteins

**Figure 7:**
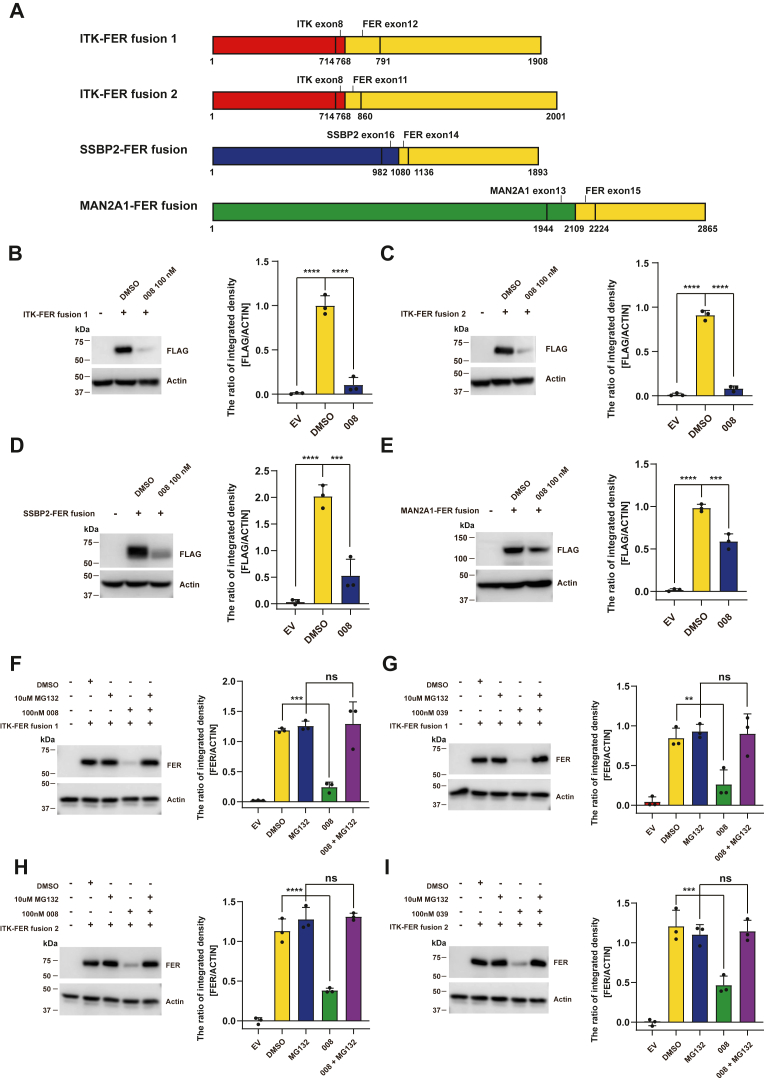
**PROTAC compounds degrade oncogenic FER fusion proteins.***A*, schematic illustration of FER fusion proteins. *B*–*E*, after transient transfection with pCDH-ITK-FER-FLAG (*B* and *C*: two fusion forms), pCDH-SSBP2-FER-FLAG (*D*), or pCDH-MAN2A1-FER-FLAG (*E*), respectively, HEK293FT cells were treated with 100 nM compound 008 for 16 h, followed by immunoblotting analysis with antibodies against FER, FLAG, and actin. *F*–*I*, HEK293FT cells transfected with pCDH-ITK-FER-FLAG (two fusion forms) were treated with proteasome inhibitor MG132 in combination with 100 nM compounds 008 or 039 for 4 h, as indicated. Cell lysates were harvested and immunoblotted with an FER antibody, using actin as a loading control. These Western blotting experiments were repeated three times. Results represented means ± SD from three replicates. ∗∗*p* <0.01; ∗∗∗*p* < 0.001; and ∗∗∗∗*p* < 0.0001. FER, Fps/Fes Related; HEK293FT, human embryonic kidney 293FT cell line; ITK, interleukin-2-induced T-cell kinase; MAN2A1, mannosidase alpha class 2A member 1; PROTAC, PROteolysis TArgeting Chimera; SSB, single-stranded DNA binding protein 2.

Alike known oncogenic fusion *ITK* (interleukin-2-induced T-cell kinase)-spleen tyrosine kinase in peripheral T-cell lymphomas, *ITK–FER* has also been identified as another potential oncogene in peripheral T-cell lymphomas by paired DNA and RNA next-generation sequencing analysis (Fig. 7*A*, ITK–FER fusion 1) (37). The ITK–FER protein retains the ITK N-terminal pleckstrin homology and FER C-terminal kinase domains (37). A very similar *ITK–FER* fusion mutation (Fig. 7*A*, ITK–FER fusion 2), caused by t (1, 5) (p34; q21.3) chromosome rearrangement, was also reported in a patient suffering from the follicular T-cell lymphoma (38). In addition, the *SSBP2* (single-stranded DNA binding protein 2)*–FER* fusion (39) and the *MAN2A1* (mannosidase alpha class 2A member 1)*–FER* fusion have also been identified in leukemia and hepatocellular carcinoma patients (40), respectively (Fig. 7*A*). Given the efficient degradation of FER protein by the PROTAC compounds, we attempted to test whether 008 and 039 could disrupt these oncogenic fusion proteins.

We constructed vectors for *ITK–FER*s, *SSBP2–FER*, and *MAN2A1–FER* and expressed these fusion proteins in HEK293FT cells, followed by 008 or 039 treatment. The immunoblotting analysis indicated that FER-targeted PROTAC could degrade the overexpressed FER fusion proteins (Fig. 7, *B*–*E*). MG132 treatment inhibited the degradation of FER fusion protein mediated by 008 and 039 (Fig. 7, *F*–*I*), suggesting the necessity of proteasome in FER fusion protein clearance.

## Discussion

In this study, we adopted protein degradation technology to design PROTAC compounds that target non–receptor tyrosine kinase FER and conduct in-depth activity evaluation and mechanism exploration on candidate compounds, laying a foundation for developing the PROTAC drugs against this oncogenic kinase. First, we verified that the Food and Drug Administration (FDA)–approved drug brigatinib (Fig. 1*A*) inhibits the kinase activity of FER (Fig. 1, *B* and *C*), which is an ideal warhead for designing FER-targeting PROTAC (Figs. 1*A* and 2, *A*–*F*). The DC_50_ values of PROTAC compounds 008 and 039 were less than 1 nM (Fig. 3, *A*–*D*), requiring E3 ligase and the proteasome machinery for their function (Fig. 4, *E*–*K*). Quantitative proteomics analysis confirmed that FER protein, along with AAK1 and GAK, as the major kinase targets degraded by the PROTAC compound (Fig. 4*D*). Through a series of functional assays, we verified that 008 and 039 significantly inhibit the motility of ovarian cancer cells *in vitro* (Figs. 5, *A*–*D* and 6, *A*–*C*) and *in vivo* (Fig. 6*D*). Finally, we demonstrated that both compounds could degrade a series of oncogenic FER fusion proteins (Fig. 7), potentially expanding the future indications for these PROTAC molecules.

Using the PROTAC strategy to degrade target proteins provides advantages over conventional small-molecule inhibitors. Take kinase-targeting PROTAC degrader as an example; it can antagonize both kinase-dependent catalytic functions and kinase-independent scaffold functions (29), thus exerting a superior efficacy. Several studies have shown that PROTAC degraders in addition suppress the kinase-independent function of FAK, resulting in better response over traditional activity–based inhibitors (29, 41, 42). Indeed, 008 and 039 showed more pronounced inhibition in the motility of ovarian cancer cells than brigatinib (Fig. 5). In alignment with previous findings revealing the kinase-independent function of FER (20, 21, 22, 23), our results collectively offer another example for taking PROTAC and other similar degradation strategies as the first choice to develop inhibitors against these kinases in the future.

To assess the selectivity and specificity of our PROTAC compounds by quantitative proteomics analysis, we found that kinases AAK1 and GAK were also efficiently degraded by 008 (Fig. 4*D*), further confirmed by biochemical analysis (Fig. 4, *E* and *F*). AAK1 positively regulates the Rabies virus entry by phosphorylating threonine 156 of the μ subunit of adaptor protein 2 (AP2M1) (43). GAK is essential for the chaperoning and uncoating of clathrin mediated by Hsc70 (44), and its kinase-dead mutant mice die shortly after birth (45). To exclude the interference of these potential targets on the cell motility phenotype observed in ovarian cancer cells, we performed another round of the wound healing experiment by evaluating the effects of our PROTAC compounds in the presence and absence of FER. The little difference in the migration ability of 008-treated CAOV4^shCon^ and CAOV4^shFER^ cells during 12 h intervals reinforced the conclusion that the suppressive effect of PROTAC compounds on cell motility was due to the on-target degradation of FER protein (Fig. 6). At longer time intervals (24 h), we did observe more profound inhibition on cell motility by 008 than FER knockdown, suggesting either incomplete suppression of FER expression by shRNA or possible attribution of degradation of other kinases by the degraders. The latter, which largely occurred in the design of kinase-targeted PROTAC compounds, should also be kept in mind during the potential translational application in the future.

AAK1 and GAK are both serine/threonine protein kinases functioning in receptor-mediated endocytosis of various RNA viruses, including rabies, ebola virus, dengue virus (DENV), hepatitis C virus, severe acute respiratory syndrome coronavirus (CoV) 1/2, and Middle East respiratory syndrome CoV (46, 47, 48, 49). A combination of two FDA-approved anticancer drugs, sunitinib (which inhibits AAK1) and erlotinib (which inhibits GAK), shows antiviral effects against ebola virus/DENV in cultured cells and also protects DENV-infected mice from death (47), supporting the feasibility of targeting both AAK1 and GAK as an antiviral approach (48, 49). However, the combined dosage required for AAK1 and GAK inhibition is not well tolerated by patients, with an increase in adverse effects primarily related to gastrointestinal disturbances (50, 51). Baricitinib, a Janus kinase 1/2 inhibitor approved by the FDA for the treatment of rheumatoid arthritis, was recently found to inhibit both AAK1 and GAK and used as a potential inhibitor for 2019-novel CoV acute respiratory disease (52). In November 2020, the US FDA approved the emergency use authorization for baricitinib in combination with remdesivir for the coronavirus disease 2019 treatment (52, 53, 54, 55). Interestingly, 008 and 039 showed robust activity in degrading GAK and AAK1 simultaneously, indicating the possible use in antiviral treatment for coronavirus disease 2019. In addition, it is worth mentioning that AAK1 is also a drug target for treating neuropathic pain (56, 57). Currently, the AAK1 inhibitor BMS-986176 is in phase II clinical trials in the United States. Its clinical indications are neuropathic pain and postherpetic neuralgia (57), suggesting another potential clinical application of our PROTAC compounds.

In summary, PROTAC compounds 008 and 039 have been designed to achieve FER degradation in a broad spectrum of ovarian tumor cell lines, thus having a significant inhibitory effect on the migration ability of ovarian cancer cells. Furthermore, 008 and 039 showed excellent degradation efficiency for the FER fusion proteins identified in various tumor samples, expanding the range for their clinical applications. This study lays an experimental foundation to apply the PROTAC strategy to antagonize cell motility and invasiveness in ovarian and other types of cancers with aberrant expression of FER kinase.

## Experimental procedures

### Cell culture and chemical reagents

HEK293FT cells and ovarian cancer cell lines (OVCAR-5, CAOV4, CAOV3, IGOV-1, PA-1, COLO-316, SK-OV-3, OVK-18, and A2780) were cultured in Dulbecco’s modified Eagle’s medium (DMEM, Thermo/Life/Invitrogen; catalog no.: C11995500CP) supplemented with 10% fetal bovine serum (FBS, PAN-Seratech, catalog no.: P30-3302) and 1% penicillin/streptomycin (TransGen Biotech, catalog no.: FG101-01). Bel7404 WT and Bel7404 CRBN-KO cell lines were gifts from Prof Yong Cang (ShanghaiTech University)’s laboratory. They were cultured in RPMI1640 medium (Thermo/Life/Invitrogen; catalog no.: C22400500CP) supplemented with 10% FBS and 1% penicillin/streptomycin. Cells were maintained at 37 °C in 5% CO_2_.

TAE684 (S1108) and brigatinib (S8229) were purchased from Selleck and dissolved in DMSO (Yeasen; catalog no.: 60313ES60) for storage. PROTAC molecules SIAIS352008 and SIAIS262039 were synthesized according to the patent (reference no.: CN202010529333).

### Cell transfection

We followed the manufacture protocol of Mirus (TransIT-2020, Mirus Bio, MIR 5405) to perform transient transfection. Briefly, HEK293FT cells were seeded in 6-well plates 24 h before transfection. When cells reached ∼90% confluence, we prepared Mirus and plasmid pEGFP-C1-FER complexes in Opti-MEM I reduced serum medium (Gibco) and added them to each well. After 20 h, cells were treated with PROTAC compounds for 4 h and then harvested for immunoblotting assays with anti-FER antibodies.

### Western blotting analysis

Cells were collected and lysed in lysis buffer (1% NP-40, 150 mM NaCl, 25 mM Hepes [pH 7.5], 1 mM sodium orthovanadate, and 1× cOmplete Protease Inhibitor Cocktail from Roche) at 4 °C. The Bradford assay or the bicinchoninic acid assay determined total protein concentration. The samples were separated by 7.5% SDS-PAGE and then transferred to nitrocellulose membranes. Membranes were blocked by 5% nonfat powdered milk (BBI; catalog no.: A600669-0250) in Tris-buffered saline with Tween-20 buffer (20 mM Tris–HCl, pH 7.5, 50 mM NaCl, and 0.1% Tween 20) at room temperature for 1 h and then incubated with primary antibody at 4 °C overnight. After washing with Tris-buffered saline with Tween-20 buffer three times, the membranes were incubated with the horseradish peroxidase–conjugated secondary antibodies (The Jackson Laboratory) at room temperature for 1 h, stained by Western lightning plus ECL (PerkinElmer; catalog no.: NEL105001EA) and detected by Amersham Imager 680/600.

The primary antibodies used in this study included FER (Proteintech; catalog no.: 25287-1-AP), FER (Cell Signaling Technology; catalog no.: 4268S), pY402 FER (Abcam; ab79573), actin (Sigma), AAK1 (Cell Signaling Technology; catalog no.: 79832), CHK2 (Abclonal; A2145), IRAK1 (Cell Signaling Technology; catalog no.: 4504), CRBN (Abclonal; A4722), and GAK (Proteintech; catalog no.: 12147-1-AP). The secondary antibodies were Peroxidase-AffiniPure Goat Anti-Rabbit IgG (H + L) (Jackson; catalog no.: 111-035-003) and Peroxidase-AffiniPure Goat Anti-Mouse IgG (H + L) (Jackson; catalog no.: 115-035-003).

### Protein degradation measurement

Cells were first seeded in a 6-well plate before treatment. When cell confluency reached ∼90%, PROTAC molecules were added. After indicated time intervals, cells were harvested, and samples were analyzed by immunoblotting with the corresponding antibodies. The relative quantification of protein was measured using Photoshop software (Adobe Photoshop 2020).

### Cell proliferation assay

CellTiter-Glo Luminescent Cell Viability Assay Kit (Promega; catalog no.: G7572) was used to determine cell proliferation by evaluating the number of viable cells in culture based on the quantitation of ATP. In brief, about 90 μl cell suspensions (3.0 × 10^3^ ovarian cancer cells per well) were seeded in 96-well white plates (Corning; catalog no.: 3917). About 24 h later, 10 μl prediluted PROTAC molecules were added to the corresponding well and incubated for the indicated time, after which 10 μl CTG reagent was added into the well and incubated at 25 °C for 15 min on an orbital shaker (400 rpm) to induce cell lysis. Then the samples were placed at room temperature for 10 min. Finally, the plate was read by a multimode plate reader (Molecular Devices; SpectraMax i3).

### Wound healing assay

Cells were seeded in 6-well plates and incubated at 37 °C before the assay. When cell confluence reached ∼90%, a straight scratch in each well was generated using a 10 μl sterile pipette tip. Wash three times with PBS to remove the suspended cells. Then cells were cultured in a fresh DMEM supplemented with 1% FBS in the presence of DMSO control, brigatinib, or PROTAC compounds. The images of the wound area were captured by a light microscope (Olympus; CKX41) at indicated time points, followed by the wound width analysis and wound closure rate calculation. The experiments were repeated three times. GraphPad Prism (GraphPad Software, Inc) was used for plotting the results. The wound closure rate was calculated as follows: (wound width at 24 h/wound width at 0 h) × 100.

### Transwell migration assay

The 8 μm chambers without Matrigel were purchased from Falcon (catalog no.: 353093). The cell suspensions (2 × 10^6^ cells per well) with DMSO control, brigatinib, or PROTACs were added to upper chambers with 1 ml serum-free DMEM. The 6-well plates were filled with 2 ml DMEM supplemented with 20% FBS. After incubating for 28 h at 37 °C and PBS washing, the migrated cells on the bottom side of the chamber were fixed with 5% formalin (BBI; catalog no.: E672001-0500) in PBS (MDBio; catalog no.: L00443) solution at room temperature for 30 min and stained with diluted Giemsa stain (Thermo/Life/Invitrogen; catalog no.: 10092013) with Gurr buffer (Thermo/Life/Invitrogen; catalog no.: 10582013) at room temperature for 1.5 h in the dark. 3Q tips removed nonmigrated cells on the top side of the chamber. The migrated cells were documented using the convert microscope (Olympus; IX73), and the number was an average of five random fields. GraphPad Prism was used for plotting the results.

### CHX chase assay

OVCAR-5 cells were seeded in 6-well plate 1 day before 10 μg/ml CHX (Selleck; catalog no.: S7418) treatment with DMSO or 100 nM PROTAC 008. Then cells were collected and lysed at indicated time points, followed by immunoblotting analysis.

### Cell apoptosis and cell cycle assays

CAOV4 cells were plated into 6-well plates and incubated with DMSO, 008, or 039. When cells reached 80 to 90% confluence, they were harvested and washed with PBS. Then, cell apoptosis quantification was performed by Annexin V-FITC/propidium iodide (PI) Apoptosis Detection kit (C1062; Beyotime). After adding 195 μl binding buffer, 5 μl FITC-labeled Annexin V and 10 μl PI were added and incubated for 10 to 20 min in the dark at room temperature. Cell apoptosis was immediately measured by flow cytometry analysis (LSRFortessa; Becton Dickinson). Cell cycle analysis was performed using the Cell cycle and apoptosis analysis kit (C1052; Beyotime). Cells were fixed in 70% ethanol at 4 °C overnight and rewashed with PBS. After the addition of 500 μl buffer supplemented with 10 μl RNase A (50×), cells were stained with 25 μl PI (20×) for 30 min at 37 °C. The cell cycle was measured by flow cytometry analysis (LSRFortessa).

### Whole-cell quantitative proteomics mass spectrometry

OVCAR-5 cells were seeded in a 10-cm dish 24 h before treatment. When they reached ∼90% confluence, cells were treated with DMSO or 50 nM PROTAC 008. About 16 h later, cells were washed with PBS twice, digested with 0.25% trypsin, and neutralized with 10% FBS–DMEM. After centrifugation and PBS washing, the cell pellets were lysed (50 mM NH_4_HCO_3_, 8 M urea [Thermo; catalog no.: 75826] and protease inhibitor [Roche; 05892791001]) and disrupted with ultrasonic cell disruptor on the ice for 30 s. Centrifugation separated the supernatant and precipitate, and protein concentrations were measured using Pierce Bicinchoninic acid protein assay (Thermo, catalog no.: 23225). Lysates were then incubated with 5 mM 1,4-DTT (Sigma; catalog no.: D9163) at 37 °C for 1 h, followed by 10 mM 2-iodoacetamide (Sigma; catalog no.: I1149) at dark for 45 min. Then lysates were diluted with 50 mM NH_4_HCO_3_ (Sigma; catalog no.: A6141) to reduce urea concentration. Lysates were digested at 37 °C overnight with 1:50 (enzyme mass:protein mass) trypsin (Promega; V5113). After digestion, the sample pH was adjusted to 2 to 4 with 10% TFA (Fluka; catalog no.: 14264) with the final concentration of 0.4%, then desalting using Sep-pak C18 kit according to the manufacturer's instructions. The peptide was vacuum dried, and 10 were analyzed and processed by the mass spectrometry instrument (Thermo Fisher; Q Exactive HF-X). Three biological replicates were performed for each sample. The scatter plot was drawn using GraphPad Prism.

### Animals

Mice were raised in the animal facility of the National Facility for Protein Science in Shanghai. All study protocols involving mice were approved by the Institutional Animal Care and Use Committee of ShanghaiTech University and conducted in accordance with governmental regulations of China for the care and use of animals.

### Statistics

The GraphPad Prism was used to perform all statistical analyses, including standard Student’s *t* test or ANOVA for multiple comparisons. The sample-size estimation, number of replicates, and data presentation were indicated for each experiment within figure legends. Data were shown as means ± SD. The following indications of significance were used throughout the article and displayed for each experiment in the figure legends: ns = no significance, ∗*p* < 0.05, ∗∗*p* < 0.01, ∗∗∗*p* < 0.001, and ∗∗∗∗*p* < 0.0001.

## Data and material availability

All the data obtained and analyzed during the current study were available from the corresponding authors upon reasonable request.

## Code availability

Software applications during the current study were available from the corresponding authors on reasonable request.

## Ethics approval

There were no study protocols involving human cancer tissues or mice.

## Supporting information

This article contains supporting information.

## Conflict of interest

The authors declare that they have no conflicts of interest with the contents of this article.

## Supporting information

Supporting Figure S1 **PROTAC compounds showed no effect on cell survival and proliferation.***A*–*D*, Cell proliferation was assessed in CAOV4 (*A*), OVK18 (*B*), PA-1 (*C*), or COLO316 (*D*) cells following incubation with DMSO, 008 or 039 using the CellTiter-Glo luminescent cell viability assay at the indicated time intervals. Results represented means ± S.D from three replicates. *E*–*F*, Propidium Iodide (PI) staining assay in CAOV4 cells incubated with DMSO, 008 or 039 to analyze the effect of PROTAC compounds on ovarian cancer cell cycle progression. Results represented means ± S.D from three replicates (*F*). *G*, Annexin V-FITC and Propidium Iodide (PI) double staining assay in CAOV4 cells incubated with DMSO, 008 or 039 to assess the effect of IRS4 knockout on cell survival. Results represented means ± S.D from three replicates (*H*).

Supporting Figure S2 **PROTAC compounds suppressed ovarian cancer cell motility at lower concentrations.***A* and *B*, CAOV4 cells were treated with DMSO, 008 or 039, respectively, followed by the wound healing experiment. Wound widths were measured, and wound closure rates were calculated (*A*). Results were represented as means ± S.D from three independent replicates (*B*). ∗∗∗∗*P* < 0.0001. (*C* and *D*) CAOV4 cells were treated with DMSO, 008 or 039, respectively, followed by the Boyden Chamber migration assay (*C*). Results were represented as means ± S.D from five independent replicates (*D*). ∗∗∗∗*P* < 0.0001.
