# Supplementary figures and images for: Development of the nonreceptor tyrosine kinase FER-targeting PROTACs as a potential strategy for antagonizing ovarian cancer cell motility and invasiveness
Source: J Biol Chem. 2023 May 16;299(6):104825. doi: 10.1016/j.jbc.2023.104825 (PMC10276154; doi:10.1016/j.jbc.2023.104825)

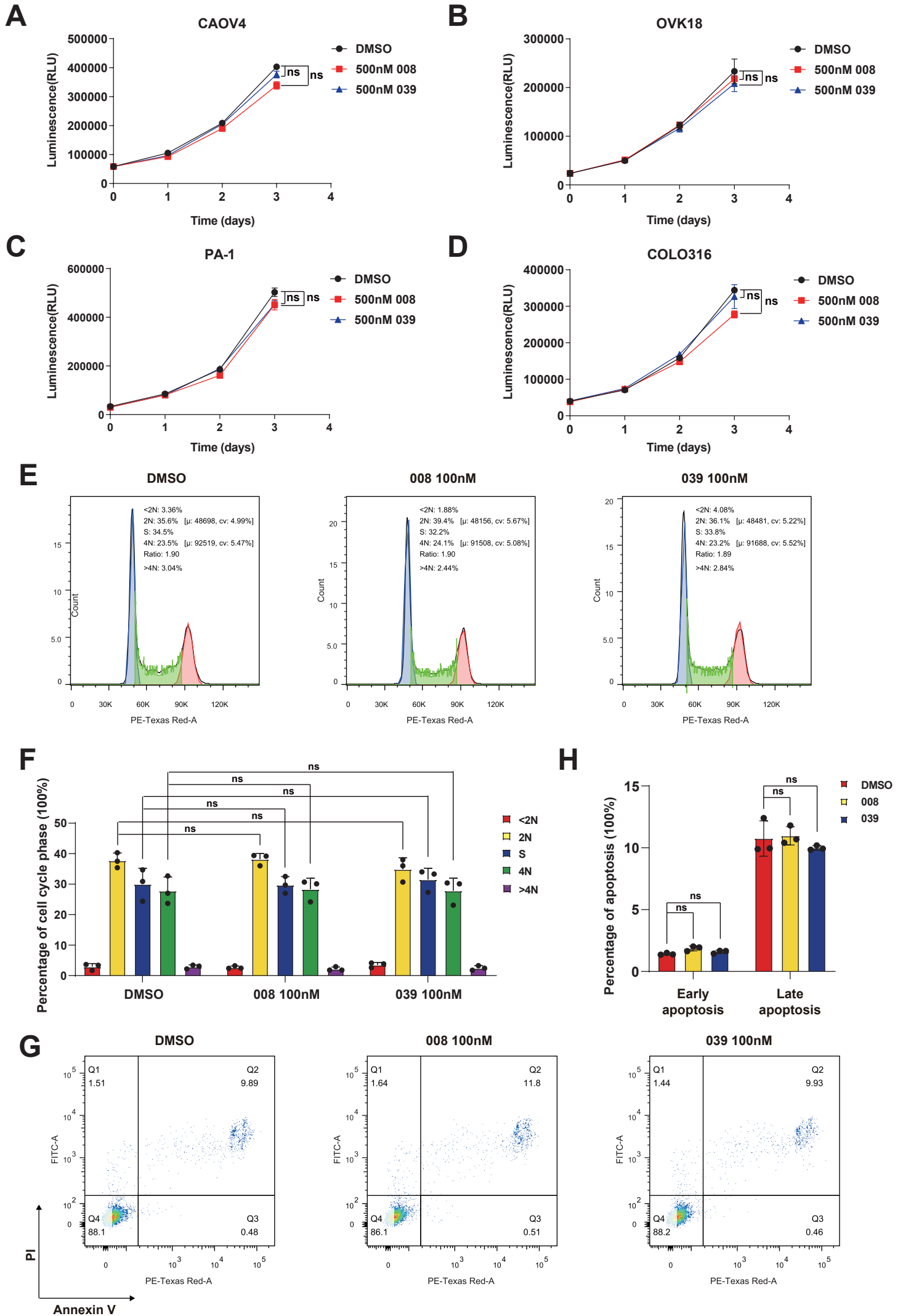

Supplement: Supporting Figure S1 — PROTAC compounds showed no effect on cell survival and proliferation.A–D, Cell proliferation was assessed in CAOV4 (A), OVK18 (B), PA-1 (C), or COLO316 (D) cells following incubation with DMSO, 008 or 039 using the CellTiter-Glo luminescent cell viability assay at the indicated time intervals. Results represented means ± S.D from three replicates. E–F, Propidium Iodide (PI) staining assay in CAOV4 cells incubated with DMSO, 008 or 039 to analyze the effect of PROTAC compounds on ovarian cancer cell cycle progression. Results represented means ± S.D from three replicates (F). G, Annexin V-FITC and Propidium Iodide (PI) double staining assay in CAOV4 cells incubated with DMSO, 008 or 039 to assess the effect of IRS4 knockout on cell survival. Results represented means ± S.D from three replicates (H). [file mmc1.pdf]

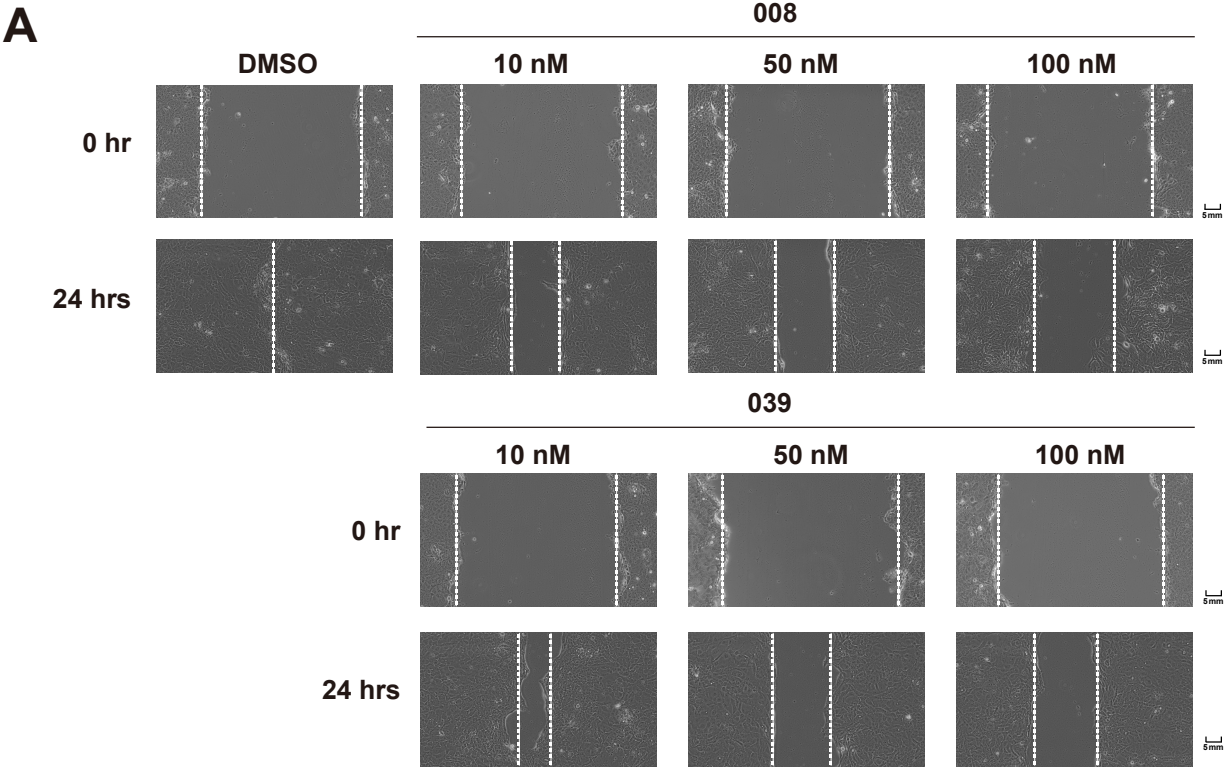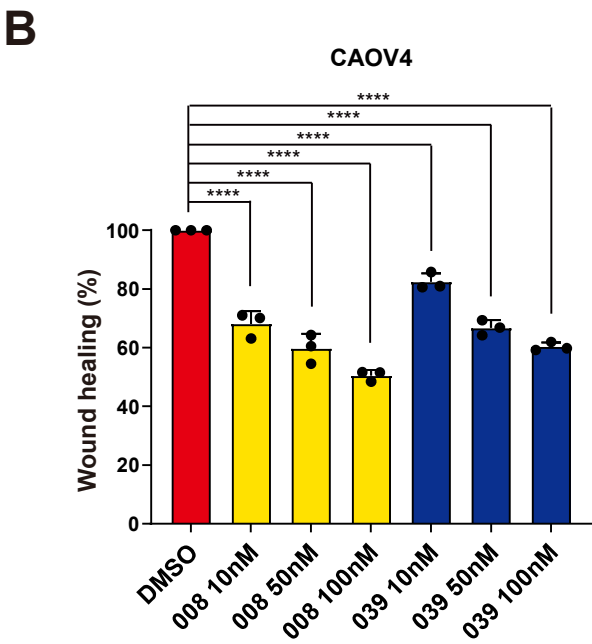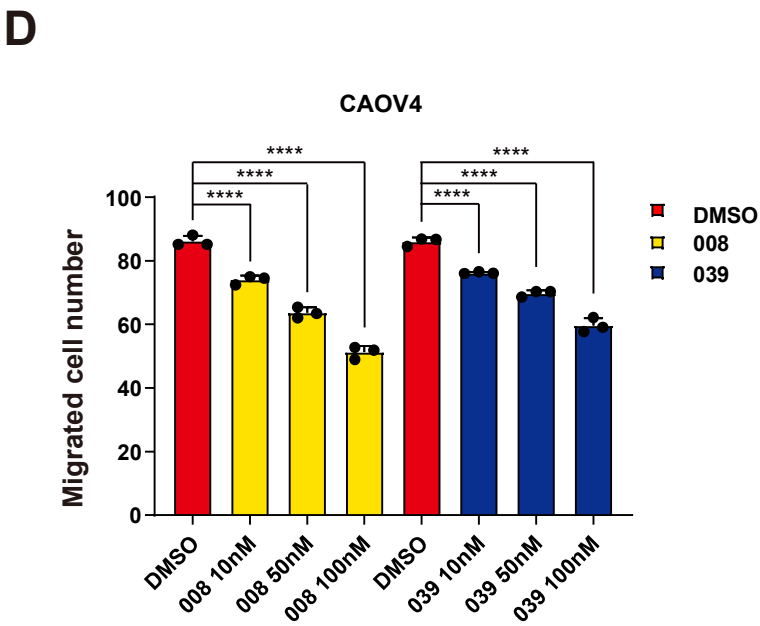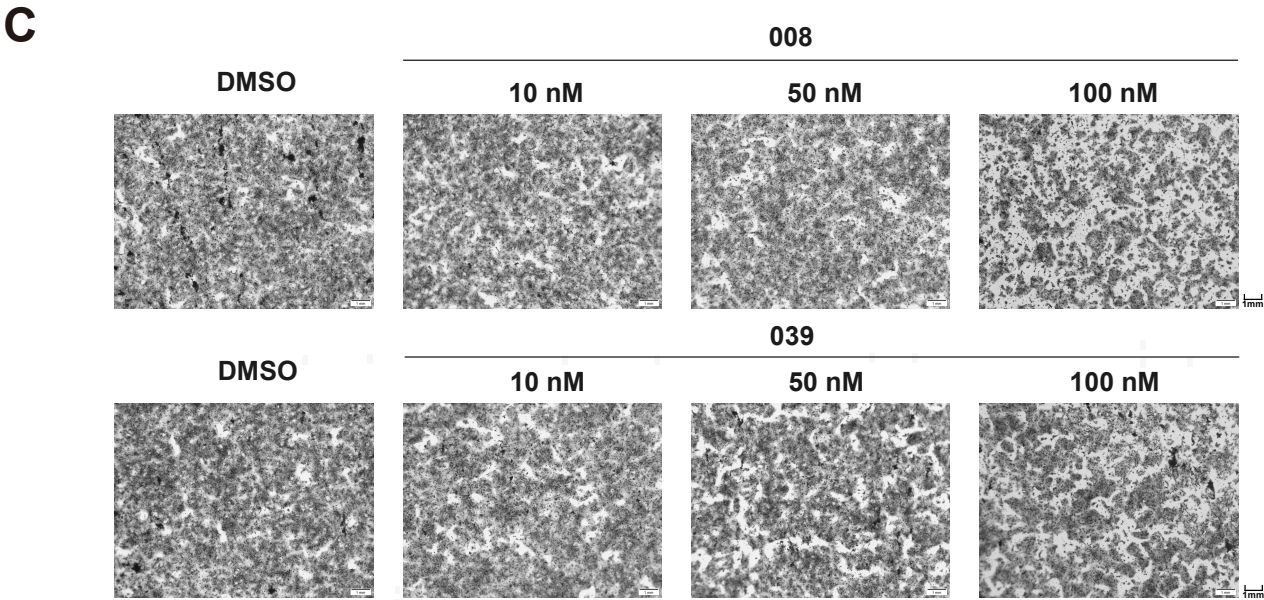

Supplement: Supporting Figure S2 — PROTAC compounds suppressed ovarian cancer cell motility at lower concentrations.A and B, CAOV4 cells were treated with DMSO, 008 or 039, respectively, followed by the wound healing experiment. Wound widths were measured, and wound closure rates were calculated (A). Results were represented as means ± S.D from three independent replicates (B). ∗∗∗∗P < 0.0001. (C and D) CAOV4 cells were treated with DMSO, 008 or 039, respectively, followed by the Boyden Chamber migration assay (C). Results were represented as means ± S.D from five independent replicates (D). ∗∗∗∗P < 0.0001. [file mmc2.pdf]
